# Applications of *Cannabis Sativa* L. in Food and Its Therapeutic Potential: From a Prohibited Drug to a Nutritional Supplement

**DOI:** 10.3390/molecules26247699

**Published:** 2021-12-20

**Authors:** Amna Iftikhar, Umaima Zafar, Waqar Ahmed, Muhammad Asim Shabbir, Aysha Sameen, Amna Sahar, Zuhaib F. Bhat, Przemysław Łukasz Kowalczewski, Maciej Jarzębski, Rana Muhammad Aadil

**Affiliations:** 1 National Institute of Food Science and Technology, University of Agriculture, Faisalabad 38000, Pakistan; amnaiftikhar445@gmail.com (A.I.); umaimarajpoot1996@gmail.com (U.Z.); 729waqar@gmail.com (W.A.); asim-shabbir@live.com (M.A.S.); 2 Department of Food Engineering, University of Agriculture, Faisalabad 38000, Pakistan; 3 Division of Livestock Products Technology, Jammu 180009, India; zuhaibbhatvet@gmail.com; 4 Department of Food Technology of Plant Origin, Poznań University of Life Sciences, 60-624 Poznań, Poland; przemyslaw.kowalczewski@up.poznan.pl; 5 Department of Physics and Biophysics, Poznań University of Life Sciences, 60-637 Poznań, Poland; maciej.jarzebski@up.poznan.pl

**Keywords:** cannabis, hemp, tetrahydrocannabinol, cannabidiol, cannabis-infused foods, cannabis products

## Abstract

Hemp (*Cannabis sativa* L.) is a herbaceous anemophilous plant that belongs to the Cannabinaceae family. The cannabis seed (hemp) has long been utilized as a food source and is commercially important as an edible oil source. In this review, the positive and negative health effects of cannabis, the relationship between cannabis and various diseases, and the use of cannabis in various food products have been discussed. In addition, the scientific literature on the potential use of cannabis and its derivatives as a dietary supplement for the prevention and treatment of inflammatory and chronic degenerative diseases in animals and humans has been reviewed. Cannabis is being developed as a key ingredient in a variety of food items, including bakery, confectionery, beverages, dairy, fruits, vegetables, and meat. Hemp seeds are high in readily digestible proteins, lipids, polyunsaturated fatty acids (PUFA), insoluble fiber, carbs, and favorable omega-6 PUFA acid to omega-3 PUFA ratio and have high nutritional value. The antioxidants of cannabis, such as polyphenols, help with anxiety, oxidative stress, and the risk of chronic illnesses, including cancer, neurological disorders, digestive problems, and skin diseases. Cannabis has been shown to have negative health impacts on the respiratory system, driving, and psychomotor functions, and the reproductive system. Overall, the purpose of this research is to stimulate more in-depth research on cannabis’s adaptation in various foods and for the treatment of chronic illnesses.

## 1. Introduction

*Cannabis sativa* L., commonly called hemp (cannabis seed) or cannabis, is the herbaceous anemophilous plant in the Cannabaceae family. Cannabis is a general word that refers to all plants that belong to the *Cannabis* genus. Most researchers are of the opinion that this plant originated in Asia and was transported to Europe as a domesticated and cultivated crop during the Bronze Age (22nd to the 16th century BC), as observed from molecular analysis, polygenetic studies, and DNA extraction from modern and archaeobotanical samples. Regardless of where it originated, *C. sativa* is widely grown and cultivated not only in Asian countries but also in Africa, Canada, Europe, and the United States [1].

Cannabis contains over 100 active chemical compounds known as ‘cannabinoids’ [2]. The plant contains a huge number of cannabinoids, the most psychoactive of which is delta-9-tetrahydrocannabinol (THC). THC also has appetite stimulant, anti-inflammatory, analgesic, and anti-emetic qualities, making it a very promising medication for medicinal applications [3]. Cannabis is used for textile and food uses since it is high in cannabidiol (CBD) or similar chemicals and is practically devoid of delta-9-THC [4]. In drug-type plants, the most abundant cannabinoids are delta-9-tetrahydrocannabinolic acid (THCA) and THC, whereas fiber-type plants are known to contain primarily cannabinoic acids, such as cannabigerolic acid (CBGA) and cannabidiolic acid (CBDA), followed by their decarboxylated forms, namely cannabigerol (CBG) ancannabidiol (CBD) [5].

There are three major species of cannabis that have been identified (sativa, indica, and ruderalis). The strength of the two main active chemicals in cannabis, THC and CBD, varies between strains, with sativa carrying the most THC and the least CBD [6]. The euphoric and psychotropic effects of cannabis are due to THC. Synthetic versions of THC, such as dronabinol and nabilone, are used to relieve nausea and improve appetite, in addition to their recreational effects. CBD, a non-psychoactive component, may be able to offset these effects. Furthermore, the Food and Drug Administration recently approved an oral CBD solution for the treatment of two uncommon, severe kinds of epilepsy as an “orphan drug.” It is frequently recommended for use in chronic pain and inflammation due to its anti-inflammatory properties [7].

**Figure 1:**
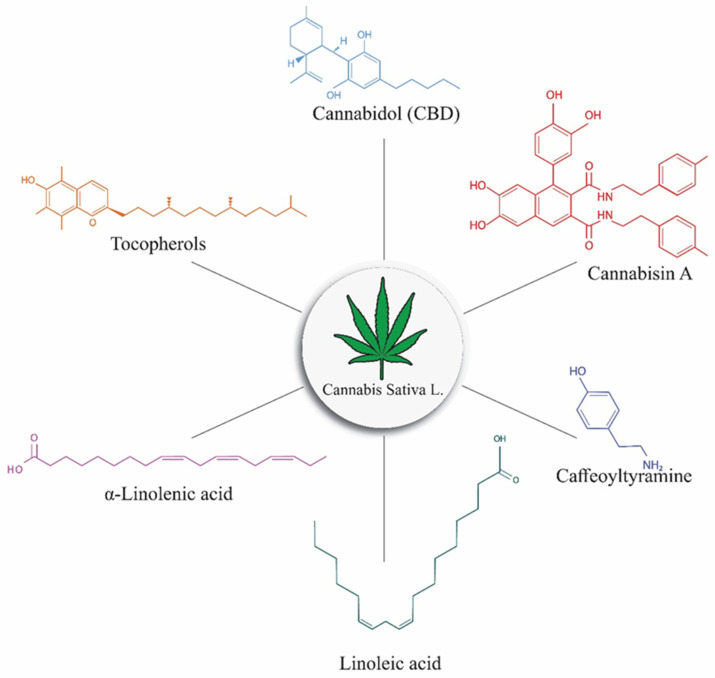
Structural components of Cannabis, including THC, CBD, cannabisin, caffeoyltyramine, alpha-linolenic acid, linoleic acid, and tocopherols.

Cannabis has a high nutritional content, which is why all parts of the plant, including the stem, seeds, roots, and flowers, have been used for food, feed, and therapeutic purposes for a long time. Hemp seed has been utilized as a food source since ancient times, particularly in Asian civilizations, and is commercially significant as a source of edible oil [1]. It consists of 30% oil and 25% protein, both of which are rich in nutritional value, as well as 10–15% insoluble fiber. Seeds can be used in cosmetics, different food products, and animal feed [8]. Seeds are subjected to cold press to extract good-quality oil. Polyunsaturated fatty acids (PUFA) abound in the oil, with an optimal ratio of -linolenic acid (ω-6) to linoleic acid (ω-3) for nutritional science (2.5–3:1). The oil is high in linoleic acid, oleic acid, stearidonic acid, and α-linolenic acid, with saturated fatty acids accounting for just approximately 10% of the total [9]. Hemp seed contains powerful antioxidants, such as polyphenols, that can help to treat many diseases, such as anxiety, oxidant stress, and the risk of chronic illnesses, including cancer, neurological disorders, digestive problems, and skin diseases. In comparison to other flowering species, the root system of hemp is well developed, which makes it ideal for the phytoremediation of heavy metal-contaminated soil [10]. Fiber residue is collected from the stem of cannabis. Hemp fiber is a highly valuable raw resource for the production of long-lasting textiles and specialized papers [1]. Hemp is composed of chemical compounds, such as linoleic acid, alpha-Linolenic acid, tocopherol, cannabidiol, cannabisin A, and caffeoyltyramine (Figure 1) [11,12,13,14]. ALA (alpha-linolenic acid) is an *n*-3 (-3) fatty acid found mostly in plant foods like flaxseed, walnuts, and vegetable oils, such as canola and soybean oils. Clinical experiments have demonstrated that substituting saturated fat with linoleic acid lowers total and LDL cholesterol, indicating that ALA has a cardioprotective effect [12]. Cannabidol, a non-psychoactive cannabinoid molecule, is a promising therapy option for both illnesses. Cannabinoids are involved in the pathophysiology of both psychotic and substance-abusing diseases (SUDs) [13]. Caffeoyltyramine and its phenolic amides, including cis-*N*-caffeoyltyramine and trans-*N*-caffeoyltyramine, are known to have anti-fungal and antioxidant effects [14].

## 2. Potential Health Benefits of Cannabis

Hemp seed is associated with a variety of health effects and possible treatments. Hemp seeds are high in antioxidants, which may help to prevent chronic diseases such as cancer, neurological diseases, immunomodulatory effects, gastrointestinal disorders, lipid metabolism, dermatological diseases, and cardiovascular health [15].

### 2.1. Cardiovascular Health

Cardiovascular disease refers to any illness that affects the heart or blood vessels. It is generally linked to the deposition of fats in the arteries (atherosclerosis) and an elevated chance of thrombosis. Cardiovascular diseases (CVDs) are the most common cause of mortality across the world. By 2030, cardiovascular disease is predicted to kill 25 million people worldwide [16].

THC was discovered to be the primary active ingredient in cannabis, which led to the discovery of specific THC receptors in the human body, known as cannabinoid receptors type 1 (CB-1) and type 2 (CB-2) (CB-2). CB-1 receptors are found in the liver, fat, muscles, and brain, whereas CB-2 receptors are found in great quantities in the immune cells, spleen, and peripheral organs, though at lower doses [17]. These findings triggered a wave of research that ultimately led to the development of endocannabinoids (ECS). ECS has been detected in cardiac tissues, and new research reveals that it is implicated in blood pressure and heart rate (HR) regulation, as well as other hereditary illnesses. In the presence of sick situations, experimental research has demonstrated redundancy in endocannabinoid signaling and endocannabinoid substrates, with dual participation of CB-1 and CB-2 receptors [18].

Steffens and Pacher [19] examined the recent literature on the cannabinoid receptor CB-2 in cardiac diseases and stated that CB-2 receptor expression in cardiac cell components, as well as attempting to penetrate immune cells, such as leukocytes and macrophages, was apparently involved in attempting to control the severity of tissue inflammation and injury occurring in different cardiovascular conditions, implying that all these receptors could play a role in controlling the severity of tissue inflammation and damage occurring in different cardiovascular disorders. The use of CB-2 receptor agonists and antagonists to pharmaceutically control CB-2 receptors seems to be a potential treatment option for conditions, such as stroke, heart failure, atherosclerosis, and restenosis. Identification of molecules with therapeutic qualities similar to cannabis and cannabinoids but with fewer adverse effects will necessitate a thorough understanding of the human endocannabinoid receptor system.

According to a recent mice study that examined three THC administration methods, pre-treatment with an ultra-low dosage of THC provided considerable protection against cellular damage to the heart, as demonstrated by reduced infarct size and low troponin levels [20]. As a result, there is data suggesting that cannabis use is linked with an increased risk of acute cardiovascular problems in both patients with atrial fibrillation and those who do not have any substantial atherosclerotic risk factors. However, some researchers feel that cannabis usage is only connected with a minimal and temporary risk of cardiovascular events, given the rarity of reports of mortality among medical cannabis users owing to the drug [21]. Some cannabis enrichment studies related to cancer are shown in Table 1.

### 2.2. Cancer

Cancer is the second biggest cause of mortality worldwide, accounting for around 8.8 million fatalities in 2015 (GHO 2018 estimates); cancer accounts for nearly one in every six deaths. Cancer is a multiphase disease that begins with the creation of a paraneoplastic lesion (initiation process) and develops to a malignant tumor over time. Cancer develops when the cellular reproduction mechanism becomes uncontrollable. It is a condition marked by uncontrolled, disorganized, and unwanted cell division. Cancer cells, unlike normal cells, continue to divide and develop throughout their lifetimes, thus generating harmful cells [11].

Cannabinoids have been shown to decrease tumor cell proliferation while sparing normal tissue. Glioma cells exposed to cannabis, for example, die from ceramide-induced cell death, but astrocytes are protected from oxidative stress in the same way [27]. Cannabidiol enhances THC’s anti-tumor action on glioma cells [28]. Finally, in certain cancers, the expression of CB1 and/or CB2 receptors has been linked to prognosis. Increased expression of CB1 and CB2 receptors has been associated with enhanced prognosis in hepatocellular carcinomas, while higher expression of CB2 receptors has been linked to higher tumor grade in gliomas [29]. Glioblastoma multiform, prostate, breast thyroid, colon, colon, pancreatic, leukemia, and lymphoma models have all shown tumor growth inhibition in vitro and in vivo. Reduction of proliferating cell signaling pathways, blockage of cell migration and angiogenesis, encouragement of apoptosis, and/or regulation of autophagy could all contribute to this anti-tumor effect [30].

THC’s safety and effectiveness in patients with refractory glioblastoma multiform were investigated in a study. Nine patients had a tumor debulking operation followed by the insertion of an infusion catheter into the resection cavity. THC was injected into the cavities every day for 10–64 days at doses ranging from 0.80 to 3.29 mg total. The drug produced a slight psychoactive effect on one patient, but it was otherwise well tolerated. THC inhibited tumor growth on MRI, as well as tumor ki-67 immunostaining and angiogenesis in tissues taken after treatment [31]. To properly identify cannabis’s potential as an anti-cancer drug, more preclinical and clinical research is needed. 

### 2.3. Disorders of the Central Nervous System

Central nervous system (CNS) illness is a wide term for a group of diseases in which impaired brain function restricts one’s health and capacity to operate. Every year, an approximate 6.8 million individuals eventually die from neurological disorders [32]. Some of the symptoms linked with neurological illnesses, such as multiple sclerosis (MS) and chronic pain, have been shown to be relieved by cannabis. MS is an autoimmune neurodegenerative disease characterized by demyelinating CNS pathology. Symptoms of this condition include spasticity, tremors, chronic pain, muscle cramps, and vesicle and intestinal dysfunctions, all of which are thought to be caused by the demyelinating process. In individuals with MS and central pain, cannabis-based medicines decreased subjective measures of MS symptoms and improved appetite [33].

Epilepsy is a neurological illness that frequently results in seizures, which are electrical discharges between brain cells that cause uncontrollable muscular activity and can also result in death. Epilepsy affects around 50 million people globally, according to the World Health Organization; the Centers for Disease Control and Prevention reports epilepsy affects 3.4 million people in the United States [34].

In 2015, the American Academy of Neurology presented a three-month clinical research study involving 137 children and young adults with various kinds of epilepsy who were treated with the CBD medication Epidiolex. Dravet syndrome (16%), Lennox–Gastaut syndrome (16%), and ten additional types of epilepsy were present in the individuals (some among them were very rare conditions). Nearly half of the individuals in this trial reported a decrease in seizure frequency. Tiredness, diarrhea, and a loss of appetite were all reported as adverse effects by 21%, 17%, and 16%, respectively. Severe side effects have been reported in a few cases, but it is unclear if these were caused by Epidiolex. There were ten cases of status epilepticus, three cases of diarrhea, two cases of weight loss, and one instance of liver injury [35].

Bergamaschi et al. [36] described long-term research in which an adolescent with significant antipsychotic side effects was given up to 1500 mg of CBD per day for four weeks. Her symptoms improved, and there were no side effects. Bergamaschi et al. reported a similar beneficial effect in another trial, in which three patients were given a starting dose of CBD of 40 mg, which was gradually increased to 1280 mg/day for four weeks.

Lorenzetti and his associates compared the neuroanatomical correlates of regular cannabis use to non-use in a meta-analysis [37]. Cannabis usage was linked to smaller brain sizes in areas involved in learning, reward, and addiction (i.e., hippocampus and orbitofrontal cortex). Neuroscientific theories of addiction have identified these pathways, and their modification has been demonstrated in substance use disorders other than cannabis. More research is needed to determine who is most prone to these brain changes, the role of vulnerability factors (such as age and sex, cannabis dependency and potency, and comorbid mental health and drug use), and how brain changes translate to cannabis-related disorders.

### 2.4. Rheumatoid Arthritis

Rheumatoid arthritis (RA) is a long-term autoimmune disorder that leads to inflammation of the joints and surrounding tissues. RA primarily affects the joints, which are generally attacked at the same time. In the years 2007–2009, about 50 million (22.2 percent) adult Americans over the age of 18 were diagnosed with arthritis, the most common of which were osteoarthritis and the autoimmune disease rheumatoid arthritis. By 2030, the initiative is expected to grow to 67 million people [38].

After employing Freund’s adjuvant to induce arthritis in rats, several CBD doses (0.6, 3.1, 6.2, or 62.3 mg/day) were given daily in a gel for transdermal administration for four days. CBD has been shown to lessen joint swelling and immune cell infiltration. After four days of CBD administration, the synovial membrane thickened and nociceptive sensitization/spontaneous pain increased in a daily dosage manner. In the dorsal root ganglia (TNF alpha) and spinal cord, pro-inflammatory indicators were similarly decreased in a dose-dependent manner (CGRP, OX42). There were no side effects, and exploratory behavior was unaffected (unlike 9-THC, which produced hypo locomotion) [38]. A cannabis enrichment study related to arthritis is shown in Table 1.

### 2.5. Dermatitis and Skin Disorders

Dermatitis is a term that refers to a group of skin conditions that cause inflammation. These skin ailments include atopic dermatitis (eczema), seborrheic dermatitis (dandruff), and contact dermatitis. Red rashes, dry skin, and itching are some of the symptoms of these diseases. Eczema (atopic dermatitis) is a skin disorder that causes redness and itching. It is most prevalent among children, although it can affect anyone at any age. Atopic dermatitis is a long-term (chronic) condition that flares up from time to time. It is possible that it will be linked with asthma or seasonal allergies. Eczema is a skin ailment that affects up to 10% to 20% of the world’s population [39].

Hempseed oil is a good source of ω-6 and ω-3 PUFAs. In a 20-week controlled, single-blind randomized trial with atopic sufferers, dietary hempseed oil and olive oil were compared. In this research, fatty acid profiles in plasma triglycerides, cholesteryl, and phospholipid components were investigated. Further information about skin dryness, irritation, and the use of dermatological medicines was obtained using a patient survey. TEWL (Trans Epidermal Water Loss) was also quantified on the skin. Both essential fatty acids (EFAs), ALA (18:3n3), and linoleic acid (18:2n6), as well as gamma-linolenic acid (GLA; 18:3n6), were enhanced in all lipid components after hempseed oil, but not arachidonic acid (20:4n6). After hempseed oil treatment, intra-group TEWL values reduced (p50.074), skin roughness and itchiness improved (p50.027), and topical medicine use was reduced (p50.024). Hempseed oil consumption led to a significant alteration in plasma lipid content, as well as a decrease in clinical signs of dermatitis. These advantages are thought to be attributable to hempseed oil’s well-balanced and plentiful supply of PUFAs [26]. A cannabis enrichment study related to skin diseases is shown in Table 1.

### 2.6. Sleep Disorders and Mental Health

Sleep is a vital physiological activity that contributes to the restoration of processes that are necessary for proper everyday function [40]. Appropriate sleep health is influenced by a number of factors, including duration, timing, efficiency, and a sense of restorative sleep that keeps a person alert and productive throughout the day [41]. Insufficient sleep is reported by 30–35 percent of the general population [42].

Cannabinoids (nabilone) were tested especially for the treatment of sleep difficulties in two studies (5 reports; 54 participants). The first was a high-risk-of-bias parallel-group trial. This study found that nabilone had a larger benefit on the sleep apnea/hypopnea index than placebo (mean difference from baseline, 19.64; *p* value = 0.02). The other was a minimal crossover experiment in fibromyalgia patients that compared nabilone to amitriptyline. This indicated that nabilone was linked to decreased insomnia (mean difference from baseline, 3.25 (95 percent CI, 5.26 to 1.24) and increased sleep restfulness (mean difference from baseline, 0.48 (95 percent CI, 0.01 to 0.95). Sleep was also investigated as an endpoint in 19 placebo-controlled studies for additional purposes (chronic pain and MS). Thirteen studies looked at nabiximols, one looked at nabilone, one looked at dronabinol, two looked at THC/CBD capsules, and two looked at smoked THC (one at various doses). Two of the trials that looked at nabiximols also looked at oral THC, and the dronabinol trial looked at oral THC/CBD. There was some indication that cannabinoids could help these patients sleep better. Cannabinoids (mostly nabiximols) were linked to a higher average improvement in sleep quality (WMD, 0.58, 95 percent CI, 0.87 to 0.29; 8 trials) and sleep disturbance (WMD, 0.26, 95 percent CI, 0.52 to 0.00; 3 trials). THC/CBD was tested in one experiment, while nabiximols were tested in the others; the results were comparable for both cannabinoids [33].

Hoch and colleagues looked at randomized clinical trials that looked at the efficacy and safety of cannabis-based medicine in the treatment of mental illnesses. In dementia, cannabis and opioid dependence, schizophrenia, general social anxiety, post-traumatic stress disorder, anorexia nervosa, attention-deficit/hyperactivity disorder, and Tourette’s disorder, their systematic review found some evidence that THC or CBD given as adjunctive treatment to other pharmacotherapies and psychotherapy improved specific symptoms of some disorders (e.g., in dementia, schizophrenia, cannabis and opioid dependence, general social anxiety, anorexia nervosa, post-traumatic stress disorder, and Tourette`s disorder). There were some negative effects mentioned, but they were rarely serious [43].

## 3. Negative Health Impacts of Cannabis

Cannabis has a wide spectrum of psychotropic effects, including mild euphoria, relaxation, temporal dilation, sensory changes, and a general pleasant feeling [44]. Cannabis has a low toxicity risk when compared to other psychoactive compounds, as animal studies have shown that the amounts required to cause death are far higher than those eaten by humans [45]. Anxiety, panic reactions, cognitive changes, and psychotic symptoms are some of the most common acute negative effects of cannabis on psychological function [44]. Cannabis and THC have been shown to cause dose-related impairment in a variety of cognitive functions, including reaction speed, perceptual motor coordination, information processing, motor performance, and mem attention [46].

### 3.1. Driving and Psychomotor Consequences

Road traffic crashes (RTCs) are a leading source of mortality and injury worldwide, and the leading cause of death among children and adolescents aged 5 to 29 years old [47]. Driving is a difficult task that necessitates the coordination of visual, cognitive, and motor movements in order to be done safely. As a result, cannabis-induced deficits in driving ability pose a serious threat to road safety. Cannabis is one of the most commonly identified psychotropic compounds in RTC-affected drivers. According to several recent meta-analyses, cannabis intoxication and recent usage increases crash risk by a modest to moderate amount, with reported odds ratios ranging from 1.28 to 2.49 [48].

According to the project “Driving Under the Influence of Drugs, Alcohol, and Medicines (DRUID)” [49], co-funded by the European Commission, the percentage of positive cannabis driver traffic crashes ranges from 4% to 14%. Delta-9-tetrahydrocannabinol (THC) was found in the blood of just 1–7% of drivers who were not engaged in a traffic collision. Senna et al. [50] calculated that cannabinoids were present in 48% of blood samples taken from suspected drug-impaired drivers in Switzerland, making them the most commonly encountered illicit narcotics. A meta-analysis based on nine trials with 49,411 participants found that drivers under the effect of recent cannabis use had nearly twice the chance of a motor vehicle collision as sober drivers [51].

A two-way mixed model study with a double-blind, placebo-controlled design enlisted 24 volunteers (12 occasional cannabis users and 12 heavy cannabis users). Both groups received single doses of THC placebo and 500 g/kg THC. Between 0 and 8 h after smoking, perceptual motor control (critical tracking task), dual task processing (divided attention task), motor inhibition (stop signal task), and cognition were all tested at regular intervals (Tower of London). THC reduced the impact of critical tracking, divided attention, and the stop signal task in infrequent cannabis users considerably. Except in the stop signal challenge, where their stop reaction time rose, THC had minimal effect on heavy cannabis users’ performance, even at high THC concentrations. There were no long-term variations in performance among heavy users due to residual THC in the groups. These findings suggest that cannabis usage history has a significant impact on the behavioral response to single THC doses [52].

### 3.2. Respiratory System

Chronic respiratory diseases (CRDs) are widely acknowledged as the leading cause of death in adult populations around the world. Chronic obstructive pulmonary disease (COPD), respiratory allergies, and asthma are all preventable and treatable CRDs. According to estimates, chronic respiratory disease (CRD) kills at least 4 million individuals each year [53].

There is significant clinical evidence linking cannabis smoke to increased airway inflammation, which is similar to the effect of cigarettes. Several pathophysiological abnormalities have been found in cannabis smokers’ airways, including vascular hyperplasia, inflammatory cell infiltrates, submucosal edema, basement membrane thickening, and goblet cell hyperplasia [54]. According to clinical observations, cannabis smokers showed higher rates of chronic bronchitis symptoms, such as cough, hyperinflation, wheezing, and sputum production, compared to tobacco smokers [55]. However, population and cohort studies have been unable to show consistent evidence of cannabis smoke’s harmful influence on respiratory health in general. Additional research discovered that the respiratory symptoms were not worse than those seen in cigarette smokers, and that they were not cumulative [56]. Furthermore, a comparison of tobacco and cannabis users in the third National Health and Nutrition Examination Survey (NHANES III) indicated that cannabis use was linked to chronic bronchitis symptoms, such as coughing on most days, phlegm production, chest sounds without a cold, and wheezing. Cannabis smoking has some notable similarities to tobacco smoking in terms of its influence on respiratory health [57]. A cannabis enrichment study related to respiratory diseases is shown in Table 1.

### 3.3. Reproductive Effects

Animals with cannabis in their systems during pregnancy have a lower birth weight [58]. The Screening for Pregnancy Endpoints (SCOPE) trial enrolled 5610 pregnant nulliparous women at minimal risk of problems from 2004 to 2011. The investigators discovered that pregnant women who used cannabis frequently had a greater chance of spontaneous preterm birth. However, there was no increase in the risk of pre-eclampsia, prenatal hypertension, or gestational diabetes [59].

Several studies imply that cannabis is the most often used illicit substance during pregnancy, despite the fact that it has been linked to negative neonatal outcomes. THC and its metabolites penetrate the placenta easily [60] and can be detected in breast milk samples up to 6 days after the mother has used cannabis [61]. Recent evidence supports an increase in their usage [62], which is more common in the first trimester and is commonly linked to alcohol, illegal substances, and tobacco [63]. The number of studies looking at prenatal cannabis consumption and pregnancy and delivery outcomes is growing. Children exposed to cannabis in utero, for example, have lower birth weight (but no difference in neonatal length or head circumference) and require placement in the neonatal intensive care unit, according to a systematic review and meta-analyses. Furthermore, compared to women who did not use cannabis while pregnant, cannabis users had a greater risk of anemia. However, no links between prenatal cannabis use and other delivery outcomes, including stillbirth or fetal distress, have been discovered [64].

## 4. Consequences of Increased THC Content

The average THC concentration of cannabis has most likely grown over the past few decades, although the exact amount is unknown due to a lack of solid data. This condition is most likely the result of a rising market for more potent cannabis products among consistent users, as well as improved methods for producing high THC content cannabis. Higher THC concentration in users may enhance negative psychological consequences, such as psychotic symptoms, deterring some from continued use. Increased effectiveness may raise the chances of developing dependency, having accidents when driving while intoxicated, and undergoing psychotic symptoms in people who continue to use cannabis [65].

## 5. Cannabis Uses

Since the legality of edibles had been proclaimed, several food corporations, manufacturers, and retailers have been looking at the possibility of marketing and selling cannabis-infused foods. In certain countries where cannabis is legalized, consumers can purchase cannabis-infused bakery products, hard candies, frozen desserts, oils, and wine. Major firms, including various beverage industries, have expressed interest in producing new culinary items that include cannabis as an ingredient [66]. The official cannabis usage database, also known as the CANNUSE database, contains information on conventional cannabis utilization from 41 countries around the world. The bulk of reports came from Pakistan (25.89%) and India (41.76%), two of the nations with the longest histories of cannabis usage in traditional medicine. The bulk of the CANNUSE database containing 2330 items (75.41%) is for medical usage, with psychoactive (8.35%) and alimentary use (7.29%). Leaf (50.51%), seed (15.38%), and inflorescence (11.35%) are the three most often utilized components of plants [67].

### 5.1. Medicinal Uses

In traditional medicine, cannabis had long been recognized as a therapeutic herb; therefore, it is not surprising that medical usage accounts for the bulk of CANNUSE database files. All plant parts have been utilized for therapeutic reasons, although leaf usage was documented in more than half of the data records (55.76%). The results of Pearson’s chi-square and Fisher’s exact tests show that some parts of the plant are not being utilized for medicinal purposes at random. They demonstrate that the leaf is closely linked to therapeutic usage. The majority of cannabis utilization for humans is in their medical applications, whereas veterinary medicine accounts for only 8.54%. Antidiarrheal usage (9.87%), dysentery therapy (6.58%), appetite stimulant (4.61%), and coccidiosis treatment (4.61%) were the most prevalent illnesses treated in animals. For usage in human medicine, they investigated 1627 human medicinal data items that were split into 16 types. Digestion and nutritional problems (17.66%), central nervous system and psychological disorders (16.24%), ache, and chronic inflammation (12.21%) were the most prevalent illnesses. They counted 210 illnesses for which cannabis was used as a therapy. The most common applications were relaxants (6.02%), analgesics (5.84%), antidiarrheal (3.01%), and anti-hemorrhoidal (2.52%), followed by dysentery (2.27%), injury therapy (2.21%), and stimulant (2.40%). Many of these applications have been tested in humans and other animal clinical trials, but the findings have been mixed or inconclusive, while others (such as anti-hemorrhoidal and wound healing) have yet to be demonstrated [68].

In the Arab world, which is another historic region for cannabis use, hashish (from the Arabic means grass) was made by compressing the resins or trichomes of the female cannabis herb. Hashish was utilized in Arab territories across the Roman Empire as a painkiller and anesthetic to treat headaches, syphilis (bacterial infection), and other medical problems. The habit of smoking only extended from European countries to the Asian and Arab World in the 16th century, owing to the introduction of tobacco from the western hemisphere. Hashish, which had previously only been consumed, began to be smoked alternatively [69].

### 5.2. Cannabis Addition in Food and Its Biological Effects

The major components liable for the biological characteristics in food items are cannabinoids, which are introduced to food matrices in high concentrations. Because THC and fat breakdown are identical, they have a lot of similarities; therefore, they may share a lot of traits. As a result, THC consumption can be determined by weight, metabolism, gender, and eating patterns. As a result, the response of each person to cannabinoids will be different. All of these variables impact bioavailability, as well as the time it takes to become intoxicated and the duration of the effects. Because cannabinoids enter the body through the digestive system rather than the respiratory tract after being taken, THC pharmacokinetics can be advantageous to the body. As a result of their lipophilic nature, fat consumption enhances THC and CBD absorption. The chylomicron converts THC and CBD into micelles for transport to the circulatory system via lymphatic capillaries after lipid breakdown in the small intestine; CB1 and CB2 receptors in the brain and immune system, respectively, connect to these exocannobinoids. Oral intake offers a longer experience than smoking and inhaling (pulmonary absorption of THC) because the most psychoactive form of THC is enzymatically formed in the hepatic system following THC metabolization. Furthermore, ingesting is less conspicuous than smoking, and it avoids the production of toxins linked with the burning of plant materials [70].

## 6. Cannabis-Infused Food Products

**Figure 2:**
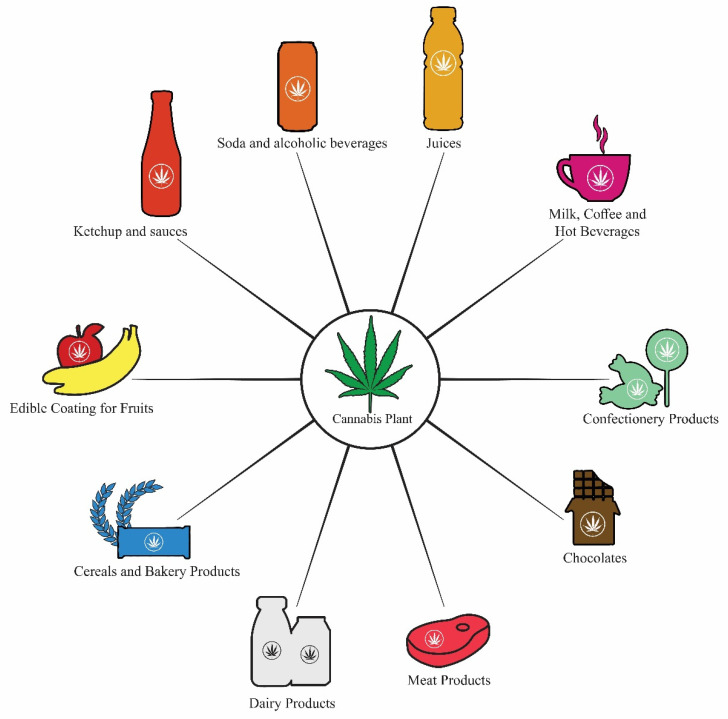
Various cannabis-infused food materials are being developed in the food and beverage industry.

Cannabis products are increasingly being acknowledged as beneficial foods. Seeds are a useful dietary source that is high in readily digested proteins, lipids, PUFAs, insoluble fiber, and carbohydrates. They have a favorable omega-6 PUFA to omega-3 PUFA ratio, are quite well suited to human nutrition, and aid in cardiovascular health, eczema, constipation, atopy, and cancers, among other disorders. Cannabis plant matter may be used to make a variety of food items. A cannabis product is a foodstuff that contains cannabis components and can be consumed as oils, oil-filled capsules, or tinctures in medicinal practice. Several cannabis-infused food items are available for recreational use. Seeds are mostly compressed for oil extraction, but they may also be found in a variety of other products, such as flavored yogurt, hemp flour, baked products, hemp milk, protein seed powder, and flavoring sauce, as well as energy bars, pralines, and chocolates, among others (Figure 2). Even though cannabis seeds and products are largely used in the modern food business, cannabis flowers, leaves, and sprouts are also consumed raw in juices or salads. Important bioactive chemicals, such as polyphenols and cannabinoids, are added in food products that are not present in seeds or are found in smaller amounts [71].

Cannabis’s alimentary usage accounts for 7.29% of all uses in the global CANNUSE database, with 58.72% of them corresponding to traditional meals and 41.28% to traditional beverages. Seeds (43.60%) are the most commonly utilized plant part for alimentary purposes. Because seeds offer abundant easily digested protein and nutritional roughage, they are still regarded as an excellent food source for older people across Asia. According to the findings, seeds are typically crushed for oils (17.65%) and pickles (14.71%). Researchers also found descriptions of them being used in drinks, as a seasoning, and as a roasted or curd-like product. Cannabis leaves are the second most commonly consumed vegetative part (37.18%), primarily in traditional drinks (such as bhang, 60.34%), and also fried or used in other recipes. The worldwide cannabis industry is estimated to be worth 20.5 billion dollars in 2020, rising to 90.4 billion dollars by 2026. Furthermore, there is also a prediction that the cannabis-infused food and beverage industry will increase by USD 22.18 billion between 2020 and 2024, marking a composite annual growth rate of 22% throughout this period, putting cannabis-based food items in an outstanding position [72].

### 6.1. Cannabis Preparation for Incorporation in Edibles

Cannabinoid extraction, separation, and refining, as well as analytical techniques for measuring cannabinoid concentration in food (cannabinoid identification and quantification), are all components of cannabis use in the food sector. The extraction process involves isolating the components of cannabis plants into an extract that can be used in a variety of ways, such as culinary ingredients, vaporization, or topical applications. Pressurized liquid extraction, solid-phase extraction, matrix solid-phase dispersion, and microwave-assisted extraction are the most frequent extraction procedures for cannabis since they are lipophilic in nature [73].

For use in foods, cannabis can be processed in a variety of ways, such as extraction of cannabis into oil for inclusion in chocolate, which requires soaking plant material to form a lipid-based formulation. Since the cannabis component would not be concentrated, the amount of cannabis that could be added to solid chocolate would be restricted by the fat’ s solution reaction. These lipids cause chocolate butter to melt at a lower temperature, resulting in soft chocolate bars that melt in the palm of the hand and lack the luster, snap, and mouthfeel of properly tempered chocolate bars. Cannabis concentrates, such as waxes, budder, shatter, and live resin, are made from the plant material. They can contain up to 90% THC in their decarboxylated state. As they are fat-based, they can be used in chocolate butter or directly in the confectionery to produce bars [74].

### 6.2. Chocolate Products

Fine chocolate contains cocoa solids and sugar. It may also contain dried milk, vanilla flavor, and other additives, depending on the recipe. White chocolate contains the same components as dark chocolate; however, it does not include cocoa solids. Tempered chocolate is used to manufacture chocolate bars, to fill shells for filled chocolates, and to coat an object in chocolate (also known as pre-crystallization). When the ideal cocoa butter crystals form during tempering, the chocolate hardens with the luster, snap, and mouthfeel that is considered perfect chocolate by consumers. Cannabis-enriched chocolate’s case study is shown in Table 2. Chocolate has an extremely low water content. When a small quantity of water is introduced to chocolate, the result is an uncontrollable hardening known as seizing. This happens as a result of wetting the sugar molecules by water in melted chocolate, causing agglomeration that does not stay in suspension any longer, which renders the chocolate useless. To be miscible with the chocolate, cannabis must be introduced in a fat-based form. Chocolate will seize if it is exposed to alcohol or glycerin tinctures, as previously stated. When approximately 20% water is introduced into the chocolate, the bulk of the sugar dissolves and the chocolate starts flowing. This means that fillings like ganache, a chocolate-cream mixture, can be prepared for bonbons. A tincture might be utilized in this situation to integrate cannabis into the product. A ganache or other filling may also be made with cannabis-infused dairy butter. However, making a ganache filling with low enough water activity to keep the solid bar’s longer shelf life is challenging [75].

### 6.3. Beverages

Plant-based meals and drinks have gained popularity over the previous decade, and the industry is rapidly expanding. For example, milk substitute beverages derived from a plant-based material that includes soy, coconut, almonds, and cannabis, such as Bjorg^®^, Evernat^®^, and Pacifc^TM^ food products, have gained prominence. Cow’s milk intolerance, such as lactose intolerance, cow’s milk allergy, such as milk protein allergies, and traditional distinctions or food preferences, such as vegetarianism, a flexitarian diet, and so on, might all be factored in the development of a market for plant-based milk alternatives. Cannabis milk is a common vegetarian substitute for cow’s milk, and it is perfect for individuals who have a milk allergy (lactose intolerance) or for individuals who avoid dairy, soy, or gluten. It is also suitable for vegetarians and vegans. Hemp milk may be readily made at home by combining water and hemp seeds. High-quality plant protein, good fats, and essential minerals are abundant in this milk. The protein level of the hemp-based milk substitute is 0.83 g/100 mL. This hemp milk also has alpha-linoleic acid, an important omega-3 fatty acid, in the amount of 0.4 g/100 mL, or 25% of the necessary daily requirement of 1.6 g. Several commercial types are also vitamin and mineral enriched. Hemp milk has less calories, protein, and carbs compared to whole cow’s milk, but almost the same amount of fat. It has a creamy smoothness and an earthy, nutty taste. It may be used in smoothies, coffee, and cereal as a replacement for cow’s milk. Hemp milk is ideal for manufacturing cappuccinos, lattes, and other coffee beverages because of its creamy texture and high protein content. People are concerned that hemp milk potentially contains THC as an ingredient, which is not the fact from a regulatory perspective. Protein blends or shakes, infusions, hemp-infused beers (e.g., Turn^®^, Cannabia^®^, Mandrin^®^, Coors Light^®^, and Appenzeller Hanfblüte^TM^), hemp-infused wines, hemp cocktails (e.g., Hempfy tonic gin), alcohols (hemp seed used as a flavoring), lemonades, Hampfy Martini, tea, (e.g., HempTea), and coffee nog are some of the hemp-based products. All of these items have a niche market focused on organic foods and drinks, as well as specialized food stores [76]. Cannabis enrichment in various beverages is shown in Table 2.

### 6.4. Gluten-Free Crackers

A functional gluten-free food application has been invented to give customers more options in this industry for value-added items. This experiment aimed to combine extremely nutritious hemp flour (a by-product of cold-pressed hemp oil) with caffeine-free green tea leaves in a portable snack-type cracker. All samples with supplemented hemp flour showed considerably better nutritional features than the brown rice flour crackers in terms of protein, crude fibers, minerals, and EFA properties. Brown rice flour crackers were supplemented with up to 30% concentration of hemp flour and 8 g of green tea leaves to increase total proteins, fibers, EFAs, mineral content, and total phenolic and antioxidant activity in comparison to brown rice flour crackers, resulting in a value-added, healthy, and nutrient-dense snack. The enrichment resulted in a substantial modification in the produced sample’s physicochemical characteristics, as well as the sensory evaluation of the panel. The samples with the highest sensory scores (8.8 and 9.0) were no. 1 (addition of hemp flour 10% and green tea leaves of 6 g) and no. 2 (addition of hemp flour 20% and green tea leaves of 4 g). There was an increase in total fibers (by around 43% and 78%, respectively) and protein content as compared to brown rice flour crackers (by around 40% and 87%, respectively). The addition of caffeine-free green tea leaves to samples 1 and 2 resulted in very strong radical scavenging (antioxidant) activity of 33.67 ± 1.32 μmol TE/g d.w. and 31.19 ± 2.57 μmol TE/g d.w., respectively. For the ideal cracker formulations, a concentration of 20% hemp flour and 4 g caffeine-free green tea leaves would be added to the dough mixture [82]. Cannabis enrichment in gluten-free crackers, a case study, is shown in Table 2.

### 6.5. Pasta

Teterycz et al. [85] fortified the pasta with cannabis flour. For this purpose, 5–40% of commercially available cannabis flour was added to the pasta. The addition of cannabis raw material to the pasta increased protein, total dietary fiber (TDF), ash, and fat content. Pasta enriched with cannabis flour at 30–40% has 19.53–28.87% d.m. of protein and 17.02–21.49% d.m. of TDF and is classified as a high-protein and high-fiber product by the European Union Commission (EU). Consumers approved the sensory characteristics of fortified pasta samples since they included safe levels of THC and CBD. The cannabis enrichment in pasta as a case study is shown below in Table 2.

### 6.6. Cookies

In cookies, different concentrations of raw and roasted hemp flour were added instead of wheat flour. The incorporation of hemp flour (raw or roasted) in cookies resulted in enhanced total phenolic content, antioxidant activity, ash, protein, and fat contents, with the highest parameters recorded at a 20% concentration. Hemp flour reduced the toughness of the cookies, resulting in softer cookies. During sensory evaluation, the cookies with 20% uncooked hemp flour and approximately 15% roasted hemp flour were assessed to be more acceptable by the panelists in terms of overall acceptability. Cannabis’ nutritional characteristics in cookie studies demonstrated that it might be used as an alternative food component in the production of functional and nutritious products [86].

### 6.7. Brownies

Wolf et al. [87] provided a procedure for manufacturing cannabis brownies and utilized them as matrix-matched calibration and quality control substances in marijuana or cannabinoid-baked edibles analysis. The manufacturing instructions for brownies, such as cake, were provided on the container of the product and were used to prepare the brownie matrix. Baked materials included five and ten mg equivalent doses/servings, or 40 and 80 ng of THC and CBD for every brownie portion (bite), respectively. The batter for brownie preparation was made and weighed according to the instructions of the manufacturers. Five and ten mg corresponding portions were obtained by weighing a 1/10 aliquant of the batter and supplementing the relevant aliquot with either 200 or 400 ng of THC and CBD. Each aliquot was well mixed with cannabis. Each fortified aliquot was divided into five wells using a dark-coated bite-sized brownie baking sheet. Then, in a laboratory oven, bake at 300 °F until a toothpick inserted into the center came out clean of uncooked batter. Cannabis enrichment in brownies is shown in Table 2.

### 6.8. Bread

Hemp flour resulted in a decrease in bread quality as well as a reduction in bread volume on baking days, as seen by its 30% proportion. As compared to wheat bread (11.02 g/100 g d.m.), hemp flour bread had a substantially greater protein content (WH50–19.29 g/100 g d.m.). The use of 30% and 50% buckwheat flour reduced the sensory evaluation of bread, particularly in terms of texture and fragrance. Wheat bread had a considerably low crumb toughness of 15.25 N on the day of baking as compared to other bread, but the hardness of bread crumb with hemp flour composition did not differ notably and ranged from 17.47 N (WH50) to 20.09 N (WH30). Wheat bread, with the largest rise in hardness and, as a result, the maximum range of the bread staling, was detected after storage, whereas the product with a 50% concentration of hemp flour had the minimum value. Breads containing 30% and 50% hemp flour showed significantly worse cohesiveness, gumminess, chewiness, and crumb resilience as compared to wheat bread and bread containing 15% hemp flour. Because of the added hemp flour proportions, fluctuations in bread crumb texture were significantly minimized. In comparison to wheat bread (73.14), hemp flour bread had a considerably reduced crumb lightness, ranging from 29.25 (WH50) to 39.91 (WH15). The higher the hemp flour concentration, the lower the value of this parameter became. In comparison to wheat bread, there was a substantial rise in redness (3.20–3.36) as the amount of hemp flour rose, as well as a significant drop in yellow (b*) pigment content (13.61–7.92) in bread crumbs (18.88 for wheat bread). On the other hand, there was a shift in the percentage of certain compounds in the polyphenol profiling, which might be related to temperature variations during baking. The addition of 15% hemp flour resulted in an increase in hydroxymethylfurfural (HMF) and furfuryl aldehyde concentration. In contrast to wheat bread, an increase in hemp flour concentration led to the reduction in HMF level, as well as aldehyde and furfuryl alcohol concentration in the trial bread [88].

### 6.9. Extruded Rice

Whole and fat-free hemp powders were mixed with rice flour at different concentrations of hemp to make extruded rice with hemp blends (0, 20, 30, and 40%). The chemical properties (carbohydrate, protein, fat, moisture, and ash) of extruded rice with hemp were significantly affected by different hemp powder concentrations for each hemp powder level. Extrudate development was significantly reduced as hemp concentrations were increased. The overall phenolic content and flavonoid content rose substantially when hemp powder was added to the solution. Extruded rice with 40% whole hemp showed the best antioxidant activity in the 2,2-diphenyl-1-picrylhydrazyl (DPPH) radical scavenging and β-carotene bleaching analyses. Moisture adsorption isotherms of extruded rice with fat-free hemp bar absorbed greater moisture than extruded rice with whole hemp bar when investigated at the same water activities. The study discovered that hemp may be used as a functional or nutraceutical component in extruded product formulations and that it may enhance the nutritional quality of food [89].

### 6.10. Gluten-Free Biscuits

Korus et al. [90] explored the qualitative, nutritional, pro-health significance, and organoleptic features of gluten-free biscuits made with acorn or hemp flour in a 20–60% maize flour replacement. Corn flour was partially replaced with the aforesaid flours, resulting in a considerable decrease in biscuit volume and an increase in biscuit toughness. The experimental biscuits were much darker in color than the control biscuits. When the amount of both flours tested was increased, a noticeable trend of color diversity appeared, ranging from yellow to red-purple. Biscuits produced with corn flour did not differ significantly from control biscuits in terms of protein content; nevertheless, biscuits made with flour hemp had 40–122% higher protein than control biscuits. Because of the inclusion of the studied flours, the total dietary fiber level rose accordingly. In comparison to the control, the total polyphenol content (TPC) of biscuits with corn flour added increased by 308–801%, while the incorporation of hemp flour boosted the TPC by 41–143%. In comparison to the control samples, corn flour contributed to a greater increase (an average of 367%) in the antioxidant activity of the biscuits than hemp flour (an average of 114%). The control biscuits, as well as biscuits with 20% and 40% acorn flour, received the highest sensory grade. Corn flour should not be substituted for more than 40% of the time with corn flour.

## 7. Safety Remains a Concern

Despite initial research indicating that cannabis is safe for consumption, this evidence from the World Health Organization (WHO) was based on acute consumption. Approximately two investigations on the safety of cannabis as a food source have now been published in the UK. A health-based guidance value (HBGV) of around 4 mg/day in a 70 kg adult was found to be safe as a conclusive result of these studies. From the Lowest Observed Adverse Effect Level (LOAEL) in humans, this would be the tolerable daily dosage. These figures are based on a modified LOAEL estimate for pharmacological treatments. The food safety authority (FSA) updated UK enterprises in 2020, restricting maximum cannabis use to 70 mg per day. Based on human liver toxicity studies, the Committee on Toxicity of Chemicals in Food, Consumer Products, and the Environment (COT) recommended a LOAEL of 4 mg/day, 105 mg on hepatic animal studies, 52.5 mg/day on animal reproductive records, while 35 mg/day on chronic animal toxicity research. As a result, the FSA’s plan goes beyond several of these suggestions, and the rationale for the FSA’s departure from COT advice remains unclear [91].

According to EU food regulation, only items that are neither harmful to human health nor unfit for human consumption could be placed on the market. Similarly, manufacturing food that can be harmful to health through the use of cannabis in foodstuffs may be actionable under UK law if levels surpass that suggested by the FSA and COT findings. The UK has given the food sector until March 31, 2021, to file a legitimate novel foods application, or items would be taken off the market. The enforcement may be based on the new food legislation or the safety of the product. A company might face legal action if it sells new foods (such as cannabis isolates) or ingredients that fall under the Public Safety Assessment (PSA’s) jurisdiction. Despite this, the FSA appears to have chosen a proportionate enforcement approach, taking into account the precautionary principle, a “likelihood of genuine damage,” the “possibility of adverse consequences,” or the presence of realistic (scientific) uncertainty regarding a product’s harmfulness [92].

## 8. Future Prospects and Conclusions

According to evidence, cannabis and cannabinoids have a wide range of biological effects, including chronic pain management in adults, antiemetics in chemotherapy-induced nausea and vomiting, and decreased patient-reported MS spasticity symptoms. Several investigations have found that they have additional health advantages, particularly as therapeutic agents. The food and beverage sectors have looked at developing cannabis-based goods as a new and innovative industry, based on evidence. The majority of the study on industrial hemp’s health benefits has been done in a preclinical phase. Furthermore, because bioactive phytochemicals can be concentrated throughout the production process, to optimize the possible health benefits while minimizing any safety hazards, the company should pay particular attention to the dose. The advantages of cannabis-derived functional food components and products, dietary supplements, and nutraceuticals on human health promotion require well-designed, randomized, placebo-controlled, double-blind research studies. Hemp seed oil has potential as a nutraceutical because of its optimal omega-6 PUFA to omega-3 PUFA ratio and bioactive cannabidiol. Polyphenols and isoprenoids, two more bioactive phytochemicals discovered in industrial cannabis, should be investigated further in the future. The impact of hemp polyphenols and isoprenoids on the sensory attributes, maintaining quality, and nutritional benefits of final products are unclear at this time. Overall, the cannabis sector is beginning to take off throughout the world. Regulatory authorities must differentiate industrial hemp from medicinal cannabis (marijuana) to fulfill the economic potential of industrial cannabis as a long-term source of value-added functional food components and nutraceutical products.

## Figures and Tables

**Table 1:** Case studies of medicinal uses of cannabis for various ailments.

Formulation and Route of Administration	Medical Condition	Cannabinoids Composition	Number of Subjects	Therapeutic Effects	Country	Reference
THC:CBD and THC extract were used	Cancer	1:1:1 of THC:CBD, THC, and placebo	177 patients	THC:CBD extract showed significant change as compared to the placebo. THC extract showed non-significant change. Induced nausea and vomiting.	Europe	[22]
CBD was used along with TRPA1 receptor, supplemented through diet	Inflammatory rheumatoid arthritis	Cannabinoids CBD and TRPA1 were used	40 patients with long-standing rheumatoid arthritis	CBD showed anti-arthritis effects, it increased the intracellular calcium levels, reduces cell viability, and IL-6/IL-8/MMP-3 production of rheumatoid arthritis synovial fibroblasts (RASF) by activating TRPA1 and mitochondrial targets.	USA	[23]
Extracts of CBD and THC was inhaled or supplemented through diet	Epilepsy	20% CBD and 1% THC were used	74 patients, aged 1–18 years	Intractable epilepsy could be effectively treated with CBD-rich medicinal cannabis. There were just a few modest and occasional adverse effects mentioned.	IL	[24]
Five different cannabis strains were used. Hybrid, Indica, Sativa, 3:1 CBD: THC, and 1:1 CBD: THC They were inhaled or supplemented through diet	Headache, migraine, arthritis, and chronic pain	3:1 ratio of CBD: THC and 1:1 ratio of CBD: THC were used	2032 patients, 62.6% male (*n* = 1271), 37.3% females (*n* = 758)	Out of a total of 2032 people, cannabis was used to treat 21 ailments. Overall, 42.4% (*n* = 861) of people had pain syndromes, with chronic pain accounting for 29.4% (*n* = 598), arthritis accounting for 9.3% (*n* = 188), and headache accounting for 3.7% (*n* = 75).	UK	[25]
Hemp seed oil was consumed orally	Dermatitis and diseases of the skin	30 mL (2tbsp) hemp seed oil and olive oil were used	20 patients with atopic dermatitis	Hempseed oil consumption resulted in significant alterations in plasma lipid content and reduced clinical signs of atopic dermatitis as compared to olive oil.	USA	[26]

CBD: Cannabidol; USA: United States of America; UK: United Kingdom; IL: Israel.

**Table 2:** Approved use of cannabis in various food products.

Product	Country	Cannabis to Product Ratio	Results	Citations
Chocolate	Canada	Cannabis was added up to 20% of the chocolate (in non-concentrated form).	The cannabis’s plant extract was effectively added to chocolate, which enhanced the physical and nutritional characteristics of chocolate without altering its qualities.	[75]
Brewing	United States	Cannabis-infused beers usually contain 10 mg of CBD and 3.5 to 6% alcohol by volume.	When people drink CBD beers, they report feeling “elevated” and “naturally relaxing.”	[76]
Tea	Italy	For 500 mL of water, 500 mg medicinal cannabis was used.	The maximum concentration of cannabinoids in the cannabis tea was obtained after 15 min of boiling.	[77]
Oolong tea	Thailand	2.5 g of Oolong tea contains 5% of encapsulated cannabis oil	Phenolics, antioxidants, flavor, aroma, and therapeutic potential were improved making the product healthier.	[78]
Yogurt	Romania	The protein content of the yogurt was increased by adding 4% cannabis protein.	Yogurt with improved nutritional, physicochemical, rheological, and sensory characteristics was produced.	[79]
Gluten-free bread	Poland	60–120 g of cannabis was utilized for substitution of 10–20% of the starch.	Cannabis enhanced the nutritional value and sensory acceptability of the bread.	[80]
Brownies	US	About 0 to 50 mg concentration of cannabis was added in brownies.	The cannabis-infused brownies were successfully produced, revealing that even the smallest cannabis dose causes discernible drug effects.	[81]
Gluten-free Crackers	Canada	20% cannabis oil press cake was utilized for the formation of gluten-free crackers.	The enrichment resulted in a substantial modification in the cracker’s physicochemical, as well as sensory characteristics	[82]
Apples-coating	Germany	Apple slices were coated with 1% pectin and 5% cannabis flour.	There was an increase in polyphenol and antioxidant capacity of apple slices and less weight loss.	[83]
Meat	Italy	50 mL of extract, containing 322.70 g/mL of CBD, was applied to 2.5 kg of meat	Cannabis extract showed antimicrobial activity against foodborne pathogens in meat.	[84]
Pasta	Italy	30–40% concentration of cannabis flour was added to pasta.	Cannabis ingredients improved the nutritional content of pasta, while maintaining its safety.	[85]
